# Relationship between Trunk Position Sense and Trunk Control in Children with Spastic Cerebral Palsy: A Cross-Sectional Study

**DOI:** 10.1155/2021/9758640

**Published:** 2021-08-19

**Authors:** Shilpa Monica, Akshatha Nayak, Abraham M. Joshua, Prasanna Mithra, Sampath Kumar Amaravadi, Zulkifli Misri, Bhaskaran Unnikrishnan

**Affiliations:** ^1^Department of Physiotherapy, Kasturba Medical College, Mangalore, Manipal Academy of Higher Education, Manipal, Karnataka, India; ^2^Department of Community Medicine, Kasturba Medical College, Mangalore, Manipal Academy of Higher Education, Manipal, Karnataka, India; ^3^Department of Physiotherapy, College of Health Sciences, Gulf Medical University, Ajman, UAE; ^4^Department of Neurology, Kasturba Medical College, Mangalore, Manipal Academy of Higher Education, Manipal, Karnataka, India

## Abstract

**Methods:**

In this study, 24 children with spastic CP aged between 8 and 15 years were recruited. They were classified based on their functional performance using Gross Motor Function Classification System (GMFCS). Trunk control and trunk position sense were assessed using the trunk control measurement scale (TCMS) and digital goniometer, respectively. The correlation between these variables was tested using Spearman's correlation coefficient.

**Results:**

Significant negative correlation was found between trunk position sense and TCMS score. Similarly, a significant moderate correlation was found between trunk position sense and GMFCS. A strong negative correlation was also found between GMFCS and TCMS.

**Conclusion:**

Children with spastic CP with better trunk position sense had better trunk control. Similarly, children with higher functional performance had better trunk control and lesser error in trunk position sense. The current findings imply the relevance of proprioceptive training of the trunk for enhancing trunk motor control in children with spastic CP.

## 1. Introduction

Cerebral palsy is a nonprogressive developmental disorder due to injury to the developing brain, characterized by abnormalities in muscle tone, movement, and motor skills [1]. Abnormalities in tone and movement are responsible for poor postural control influencing daily functional activities such as sitting, reaching out, and walking [2, 3].

The trunk forms an initial frame for postural control, during postural stabilization and orientation; hence, it plays a vital role in performing goal-directed activities [4–7]. Trunk control, an active component of postural control, is a prerequisite for adequate, free, and specific mobility of the head and extremities [8–10]. Children with spastic cerebral palsy have poor trunk control owing to the weak trunk muscle strength, altered neural control, and inadequate position sense [11, 12]. Literature review suggests a relationship between trunk control and sitting postural control, wherein children with CP present with impaired trunk control and poor maintenance of upright posture owing to impairments in anticipatory [13] and reactive [14, 15] postural responses and changes in ground reaction force during postural adjustment [16].

Topographically, trunk control is impaired significantly in quadriplegics followed by diplegics and lastly hemiplegics [2, 17]. Also, children with quadriplegia manifest impaired static as well as dynamic trunk control, whereas children with diplegia and hemiplegia usually present with only an impaired static trunk control [2]. Maintaining trunk control while performing trunk movements in the sagittal plane is relatively easier than the frontal plane movements [2, 18]. Based on the motor involvement, children in the lower Gross Motor Function Classification System (GMFCS) level had profound impairment of trunk control suggesting that there is a relationship between functional abilities and trunk control [2, 12]. In addition, impaired trunk control in children with spastic CP is associated with balance dysfunction [2, 19].

Sensory deficits in children with CP, including proprioceptive dysfunction, can influence postural control [20, 21]. The joint position sense, a component of proprioception, increases body awareness and contributes to motor control and motor planning. Trunk position sense plays a crucial role in the maintenance of normal spinal mobility and stability [22] and helps in the development of motor skills [2] and postural control [13, 23]. Altered trunk position sense has been established in various populations, such as poststroke hemiparesis people [11], balance-impaired older adults [24], and people with orthopaedic spine disease [22, 25, 26], but no studies discuss the impaired trunk position sense in children with CP.

The stabilization of the trunk is a core element to improve head stability, visual field orientation, and hand manipulation across a wide range of children with CP [27]. It is known that the trunk remains an essential component for the independent functioning of a child with a neurological disorder and children with CP have impaired selective control of the trunk affecting their ambulation and functional activities [28]. Literature review suggests several studies discussing the relationship between trunk position sense, balance, and postural control in various neurological conditions [11, 29, 30]. However, in children with spastic CP, this relationship remains mostly unexplored. Therefore, this study was carried out to determine the relationship between the trunk position sense and trunk control in children with spastic CP.

## 2. Materials and Methods

### 2.1. Participants

A total of 24 children with spastic CP were recruited from the neurosensory developmental rehabilitation unit in Mangalore city. The Institutional Ethics Committee of Kasturba Medical College (Manipal Academy of Higher Education), Mangalore (IEC KMC MLR 11-13/224), approved the study. Children with all the types of spastic CP aged between 8 and 15 years, who were diagnosed with CP by paediatricians through MRI in GMFCS levels I-III, who could sit independently for 30 minutes, without back support and feet placed on the ground, and with Modified Mini-Mental Scale Examination score not less than 24, were included in the study [31]. Children were excluded if they had hearing or visual disabilities; if they had a history of other neurological, musculoskeletal, or medical conditions; and if they have been administered neural blockers like botulinum toxin and phenol in the last six months, interfering with the performance. In addition to the above, if the children experienced any pain during trunk movements during the assessment procedure, they were excluded from the study. All the eligible participants were approached; the objectives of the study were explained to their parents/legal guardians in a language they understood. A written informed assent from the participants and consent from the parents/legal guardians were obtained.

### 2.2. Procedure

The trunk control measurement scale (TCMS) was used to measure the trunk control. The trunk position sense was measured using a digital goniometer (Human Performance Measurement, Inc. Advanced Performance Meter 1 Digital Goniometer) in a supported standing position. Both trunk control and trunk position sense were assessed in a single contact session by the same trained therapist in a random order by a coin toss method to prevent any carryover effect of one assessment over the other. In total, the assessment was completed in a duration of 35 minutes to complete the entire assessment of a child with 15 minutes for trunk control assessment and 10 minutes to assess the trunk position sense. An additional 10-minute break was given between the two assessment measures to avoid the effect of fatigue over the second assessment measure.

#### 2.2.1. Evaluation of the Trunk Control

Out of several tools available to measure trunk control, TCMS is proved to be a clinically comprehensive scale developed to assess the static and dynamic components of trunk control among children with spastic CP [17]. The TCMS comprises 15 test items measuring both static and dynamic components of the trunk control in sitting. The maximum value for the total TCMS is 58 points (20 for the category “static sitting balance,” 28 for “selective movement control,” and 10 for “dynamic reaching”). A higher TCMS score indicates better trunk control. The scale has acceptable interrater and intrarater reliability (interclass correlation coefficients = 0.91–0.99; kappa = 0.45–1; Cronbach's alpha coefficients = 0.82–0.94) and good validity (Spearman rank correlation between GMFM dimension B to E and TCMS, *r* = 0.82–0.94) for assessing trunk control in children with spastic CP from 8 years to 15 years [12, 17]. The standard technique was used to measure trunk control using TCMS [32].

#### 2.2.2. Evaluation of the Trunk Position Sense

**Figure 1:**
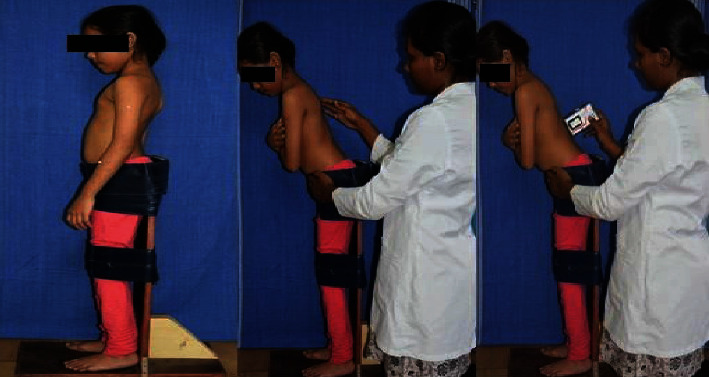
Measurement of trunk position sense.

Active position sense of the trunk was assessed using a digital goniometer. Each participant was made to stand on a custom-made wooden frame with feet in a neutral position, shoulder-width apart, knees extended (fastened with straps), hips relatively in neutral position, and pelvis strapped below the anterior superior iliac spine to decrease any proprioceptive feedback from the lower extremity and pelvis (Figure 1). The testing procedure was explained and two practice trials were given, where each participant was passively moved to the target position of 30° trunk flexion; they were instructed to hold and memorize this position for 10 seconds with eyes open and also to understand the test procedure. Once the practice trials were completed, the participant was brought to the neutral position and was instructed to replicate the target position as accurately as he or she could with closed eyes. Participant-perceived target position was measured using a digital goniometer with the reference point at the L1 lumbar spinous process.

No verbal or visual feedback or clues were given to the participants during the performance. All the participants could comply with the test without any adverse events. To add on further, considering the muscle strength impairments seen in CP, an angle of 30° was chosen to test the trunk position sense; further increase in the range would have put additional load on the muscles. Inability to reposition the trunk to a greater flexion angle because of impaired muscle performance would adversely affect the test results.

### 2.3. Statistical Analysis

The collected data were coded and entered into Statistical Package for the Social Sciences (SPSS). The results were expressed as summary measures (median and interquartile range) and proportions using appropriate tables. The correlation of TCMS, trunk position sense, and GMFCS was done using Spearman's correlation coefficient as the data was not normally distributed, which was revealed on the Shapiro-Wilk test. A *p* value of <0.05 was considered statistically significant. Correlation coefficient (*r* value) between 0.00 and 0.10 is considered as negligible correlation, 0.10 to 0.39 as weak correlation, 0.40 to 0.69 as moderate correlation, 0.70 to 0.89 as strong correlation, and 0.90 to 1.00 as very strong correlation [33].

## 3. Results

This study included 24 children with spastic CP, and their demographic characteristics are depicted in Table 1. The descriptive analysis of trunk position sense and TCMS scores, including median and interquartile range, is mentioned in Table 2.

### 3.1. Relationship between the TCMS and Trunk Position Sense

The trunk position sense showed a moderate negative correlation with the total TCMS score (*r* = −0.58, *p* = 0.003) as well as static sitting balance and dynamic selective movement control subscale of TCMS, with a *p* < 0.05 (Table 3). However, dynamic reaching, another subscale of TCMS, showed a weak negative correlation with trunk position sense (*r* = −0.35, *p* = 0.098).

### 3.2. Relationship between GMFCS, Trunk Position Sense, and TCMS

When GMFCS levels were correlated with trunk position sense, a moderate significant positive correlation was found (*r* = 0.60, *p* = 0.002) (Table 4). A strong significant negative correlation was found (Table 4) when GMFCS levels were correlated with a total TCMS score (*r* = −0.87, *p* < 0.001).

## 4. Discussion

The present study is aimed at examining the relationship between trunk position sense and trunk control in children with spastic CP. Children with spastic hemiplegic and diplegic CP have previously exhibited good trunk control in static sitting conditions as this requires the trunk to be erect during quiet sitting and when the extremities are moved [18]. They also have fairly good control in forward-reaching tasks with challenges in lateral reaching and cross rotations [2]. The present study included primarily children with spastic diplegia, and a similar presentation was observed with respect to static sitting balance and dynamic reaching subscale. Abnormalities of selective movement control have been widely recognized as a primary manifestation of CP [34]. The movements of the trunk assessed in the dynamic selective movement control subscale are multiplanar, and the aforementioned loss of selective motor control could lead to poorer performance. The results of the present study revealed that children with spastic CP exhibited poor trunk control when evaluated using the TCMS, which is in agreement with the existing literature that investigated the trunk control in children with spastic CP [2]. This is the first study that evaluated trunk position sense in children with CP and revealed errors in repositioning the trunk which is higher than the normal individuals who are found to have an error of 2.6° [35].

It was found that the individuals with impaired trunk position sense are able to perform motor tasks; however, the quality of the movement is compromised and the goal-directed actions execute lack of precision and postural responses, causing impaired balance and gait [36]. When trunk position sense was correlated with the subcomponents of trunk control, both static sitting balance subscale and dynamic selective movement control subscale showed a moderate significant negative correlation. In the static sitting balance subscale, the ability of the children to maintain stable sitting posture keeping the trunk erect when the limbs are being relatively moved is assessed, and during such a task where the body is in a stable position, the body primarily depends on the proprioceptive system for maintaining balance [37, 38]. Hence, children with better trunk position sense perform better in the static sitting balance subscale. The test conditions in the dynamic selective movement control subscale consist of items where the ability of the children to move the trunk in different planes is assessed, which again primarily relies on the proprioceptive feedback from the body to execute the task [38].

Dynamic reaching subscale which tests the child's ability to reach both sideways and forwards beyond the limits of stability showed a weak negative correlation with trunk repositioning error. This could be because such conditions involve movements that require the use of feed-forward control where the anticipatory postural adjustments are made to maintain stability [36]. In the presence of inaccurate or reduced information from the somatosensory system, this feed-forward control relies more on the vestibular system [39].

In the current study, as the functional level increased on GMFCS, the error made with respect to trunk position sense decreased. The study findings are in line with the previous findings by Han et al. in which the children with lesser proprioceptive dysfunction had shown better motor performance, necessitating the role of proprioception for motor function [40]. Marked differences were found when GMFCS levels and TCMS in total and subscale scores were correlated. As the level of impairment increased on GMFCS, i.e., from level I to level III, the trunk control decreased, indicating a strong significant negative correlation of trunk control with GMFCS. The findings mentioned above suggest that trunk control is an essential component of functional abilities in CP children. This is in agreement with a study on impaired trunk control in spastic CP children, where trunk impairment was assessed based on the severity of motor involvement, which finally concluded that more significant deficits in trunk control were found in severely impaired children [2].

Though the study provides a unique understanding of trunk control impairment and trunk position sense, some critical reflections are warranted. The children included in the study were from GMFCS level I, level II, and level III. Hence, results cannot be generalized to all GMFCS levels. Children with spastic CP should be homogeneously grouped based on topography and GMFCS levels to find the appropriate correlation with clinical implications. Future studies encompassing trunk control, trunk position sense, and trunk muscle strength may contribute to a better understanding of the functional relationship in children with CP.

## 5. Conclusion

In conclusion, the results of this study provide additional evidence of trunk control being impaired in spastic CP children. Children with spastic CP with better trunk position sense had better trunk control. Similarly, children with higher functional performance had enhanced trunk position sense and better trunk control. The present study findings also imply that therapeutic intervention focusing on proprioceptive training on an unstable surface and narrow base of support, which relay conflicting somatosensory feedback, may further improve trunk control in children with spastic CP.

## Figures and Tables

**Table 1:** Demographic data of the participants of the study (*n* = 24).

		*n* (%)
Age group (8-15 yrs)	8-10 yrs	15 (62.5)
	11-15 yrs	09 (37.5)
Gender	Male	16 (66.7)
	Female	08 (33.3)
Dominance	Right	21 (87.5)
	Left	03 (12.5)
Types of spastic cerebral palsy	Spastic diplegia	21 (87.5)
	Spastic hemiplegia-left	02 (8.3)
	Spastic triplegia	01 (4.1)
GMFCS levels	GMFCS I	12 (50)
	GMFCS II	09 (37.5)
	GMFCS III	03 (12.5)

*n*: number of subjects; GMFCS: Gross Motor Function Classification System.

**Table 2:** Trunk position sense and TCMS mean scores of children with spastic CP.

	Minimum	Maximum	Median [IQR]
Trunk position sense (degree)	1	8	4.5 [2.75, 6.00]
SSB/20	8	20	18 [15.75, 20.00]
D-SMC/28	9	25	22.00 [12.75, 25.00]
DR/10	5	10	10 [8.75, 10.00]
TCMS (total)/58	23	55	49 [35.75, 55.00]

IQR: interquartile range; SSB: static sitting balance; D-SMC: dynamic selective movement control; DR: dynamic reaching; TCMS: trunk control measurement scale.

**Table 3:** Correlation between trunk position sense and TCMS (*n* = 24).

	SSB	D-SMC	DR	Total score TCMS
	*r* (*p* value)	*r* (*p* value)	*r* (*p* value)	*r* (*p* value)
Trunk position sense	-0.57 (0.004^∗^)	-0.62 (0.001^∗^)	-0.35 (0.098)	-0.58 (0.003^∗^)

^∗^Significant. SSB: static sitting balance; D-SMC: dynamic selective movement control; DR: dynamic reaching; TCMS: trunk control measurement scale.

**Table 4:** Correlation between trunk position sense and TCMS with GMFCS levels (*n* = 24).

	GMFCS
	*r* (*p* value)
Trunk position sense	0.60 (0.002^∗^)
TCMS	-0.87 (<0.001^∗^)

^∗^Significant. GMFCS: Gross Motor Function Classification System; TCMS: trunk control measurement scale.

## Data Availability

The data used to support the findings of this study are available from the corresponding author upon request.
